# Supplementary material for: The use of audio self-hypnosis to promote weight loss using the transtheoretical model of change: a randomized clinical pilot trial
Source: PeerJ. 2022 Dec 14;10:e14422. doi: 10.7717/peerj.14422 (PMC9758970; doi:10.7717/peerj.14422)
Supplement: Supplemental Information 1 [file peerj-10-14422-s001.docx]

Appendix A: the scripts of the audio files

Hypnosis Audio file:

Hello! I am Myrna Saadeh, clinical hypnotherapist.

When listening to this recording, you should only be listening to it in the morning and at night, when you are safe, and no one and nothing will disturb you.

You will be receiving positive suggestions that will create positive lifestyle changes and help you lose weight.

Never play this recording while you’re driving. Thank you and enjoy!

1. Induction:

Please make yourself comfortable...Close your eyes and let yourself relax... Take a few, slow, deep breaths and notice that as you exhale, you can feel yourself becoming more and more relaxed. You can continue to relax, as I speak to you. And each time you exhale, you can feel yourself becoming more and more relaxed...more and more relaxed. Soon you will experience hypnosis. I want to assure you that no matter how deeply hypnotized you become, you will remain in complete control. You will stay in control, even when very deeply involved in the experience of hypnosis. I will make suggestions, and you will experience those suggestions. You will find it very easy to experience what I'm telling you than you ever thought possible. So the choice is always yours, and it’s safe to enter hypnosis now, as you allow yourself to relax more and more. .

As I speak, you can feel yourself becoming more relaxed. But no matter how relaxed you become, you will hear my voice, and you will be able to respond to my suggestions. If you become uncomfortable, or need to pause or stop for whatever reason, you can readjust your body and make yourself comfortable again, and that won’t get in the way of your experience.

Right now, you might want to relax even more. Let your body relax...Just begin to feel a spreading sense of calm...and peace...letting go of all your cares and concerns, let them drift away, like clouds in the wind.... more and more at peace…more comfortable and secure

Focus your attention on your toes...Let your right and left toes relax...relax completely...Letting your toes relax...more and more...more and more relaxed. And let the relaxation spread from your toes into your feet, and let your feet relax. Let them become more and more relaxed...as you can feel so calm and at ease.

And now pay attention to your ankles and to your calves. I wonder if you can begin to let go...let go and relax as you feel perhaps a comfortable sense of warmth in your ankles or your calves...Or perhaps it is a cool and easy feeling...in your right leg or in your left leg. Just let your legs relax...more and more relaxed...more and more completely relaxed.

And the relaxation can spread into your thighs...your thighs can relax more and more...just letting go. Relaxing more and more. Relax your stomach. Just let it go loose and limp...loose and limp. Notice how it feels. And let the relaxation spread upward into your chest. Let all the nerves and muscles in your chest relax completely...relaxed. a a deep breath in now, inhale and exhale, feel the peace spreading as you feel so at ease...so secure...your body and mind so relaxed and at peace. And now your back can relax, your shoulders. Let yourself feel the relaxation in your back and your shoulders...more and more relaxed...loose and limp...completely relaxed.

Let the relaxation spread through your arms, down into your hands and your fingers. Relax the muscles of your neck. Just let go and relax...loose and limp...completely relaxed. And relax your jaw muscles. Just let them go limp. All the nerves and muscles in your jaw relaxing completely. And relax all the rest of the muscles in your face...your mouth...nose...eyes...eyebrows...eyelids...forehead...all the muscles going loose and limp...loose and limp...completely relaxed...at peace...calm and relaxed... completely at ease.

You might like to imagine being somewhere peaceful and relaxing. I like to imagine lying on a quiet beach on a perfect day, with a beautiful blue sky and just a few billowy clouds floating by... I can imagine feeling a soft, gentle breeze...cooling my skin...smelling the salt sea air...but you can imagine being anywhere you like.

It might be someplace you’ve been...or someplace you’d like to be. Or just a place in your imagination...It doesn’t matter...all that matters is your comfort...your peace. Wherever it is, it is so peaceful and calm...someplace where you can just be you...where you can feel completely at ease and content. And you can imagine yourself actually being there...seeing, the things you would see...feeling the things you would like to feel...hearing the sounds you would like to hear...and smelling your favorite fragrances.

1. Deepening:

And while you are in your happy safe place, I am going to count from one to ten. And with each count you can drift more and more deeply into hypnosis...more and more able to experience whatever you want to experience. One...drift...drift deeper...two...always taking your deeper breaths, feeling more and more balanced... three ...four ...deeper and deeper ... five ... halfway there ...six ...seven ...even deeper than before...so deep that you can experience whatever you wish to experience ...eight ...deeper and deeper, nine ...ten ...very deep now...very deep...completely at one with yourself...deeply relaxed.

The unconscious part of your mind is allowing this Audio to make a lasting impression over the way you think, feel and behave about food and exercise.

1. Suggestions:

While you are in this special place, I would like you to experience all the things you would see...all the things you would hear...all the things you would smell...all the things you would touch...Focus on all these sensations, it is almost like you are really there now, enjoying this special place. More and more comfortable...more and more relaxed...more and more at ease...more and more deeply hypnotized. It is so perfect. With each breath, you can become more and more deeply hypnotized. So deep that you will be able to do whatever you need to do in hypnosis today...deep enough to experience anything you wish to experience.

I would like you now to focus on your special place. Just be there now, and know that you are at peace, happy, motivated calm and relaxed, yet energized. Concentrate on this feeling, and know that you can take it with you throughout your weight loss journey.

Now listen to my voice and only my voice. Listen only to me and allow your thoughts to fill with what I say. All other sounds ... which you may hear ...will have only a calming, soothing effect.

I am going to give you some suggestions that will make this a permanent change in your living. These suggestions are going to take complete and thorough effect upon the deepest part of your subconscious mind. They will remain there forever, and become a permanent part of every cell of your brain and body. You are going to be surprised and amazed just how effective these suggestions are going to be and how much they will become a part of your everyday life, giving you a brand new pattern, brand new thoughts, a brand new method of action, to make you successful in releasing all those extra kilograms.

You will be a new person, in a new form, with new eating habits. You are going to enjoy life. You will eat only when you have physiological needs for food and no other time, for the rest of your life.

From this point forward, starting right now ... you will no longer have the urge to snack between meals. All desire to eat fattening, heavy, rich foods is draining away. The inclination to eat such foods is leaving you now ... and is becoming like a distant memory. It is simply a past experience ... it has no effect on you now. You no longer have the desire or appetite to get a late night snack; because you are already full... you are completely satisfied by a normal well balanced meal. You will no longer overeat at mealtime, because moderate meals will more than satisfy your hunger. And the flavor and tastes of food will be so sharp that your appetite will be satisfied, as it has never been before. The enhanced taste of food will fill you up quickly and satisfy your hunger. The delicate flavor of each bite of food will be more enjoyable than before. You enjoy the taste and fragrance of your food more than ever before. You will feel so much better, so much healthier, so much happier and so much more energetic without an uncomfortably over filled stomach.

Each time you are tempted to eat or drink anything that you know is wrong for you, you will say “no” and stick by it, because the rewards of enjoying your meals and taking care of yourself are more important to you than eating the wrong foods.

Now imagine your MOST IMPORTANT BENEFITS SO VIVIDLY that you feel as though you already enjoy success. Imagine your attitude of gratitude … and be glad. [Slowly] Imagine your benefits … SO VIVIDLY that you FEEL as though you already enjoy SUCCESS! If you choose these benefits for yourself, then indicate that choice right now by moving one of your fingers.

You have used your power of choice to choose your benefits. The terms are so simple. You simply use that same power of choice to choose what goes into your mouth … when, where, and how much. You decide what goes into your mouth … when, where, and how much. And you have an increasing satisfaction from the right amounts of those foods which help you reach your ideal, healthiest body weight. And you make wise choices about your health and eating habits. Also, whenever you choose water or a non-caloric beverage to satisfy in between meal snack urge, you are TOTALLY satisfied – physically, mentally, and emotionally, because YOU CHOOSE …

You LOVE your power of choice. And like a muscle that’s used becomes stronger with use, your power of choice becomes stronger with use. The benefits are so satisfying and you love your power of choice.

Also, whenever you eat, you eat SLOWLY enough to … ENJOY the FLAVOR of each bite. And when you have had enough food to give your body nourishment, you are satisfied - physically, mentally, and emotionally … and every day it becomes easier for you to be TOTALLY self-motivated to do those things that help you reach your ideal body weight, because you love the benefits.

You eat … SLOWLY enough to … LISTEN to your body and FEEL your satisfaction. You allow your physical and emotional appetites to harmonize. And when you have had enough to eat, you simply take your thumb or finger and do a PUSH AWAY. Simply push your plate even so slightly away from you – pushing away the excess food and the excess kilograms – kilogram after kilogram. You release the excess food and you release the excess kilograms - kilogram after kilogram – until you reach your ideal body weight.

Eating well balanced meals will increase your energy. And with your new lighter figure, you will find that your energies have increased and you will feel stronger and healthier as you approach and achieve your ideal weight and figure. Never again will you eat because you are nervous, tense, bored, frustrated, depressed or angry.

The key to achieving your fitness goals is to get excited about it. And from this moment on you are totally excited about the new you that you are creating. You now focus on the end results of what you would like to look like and how you feel as the goal you. Visualize yourself as the goal you already and realize what it took to get you here. Excitement, exercise and motivation. You are very eager to attain this goal and each day you wait in anticipation for the moment to come that you can begin exercising knowing that you are becoming the goal you each day. It is now enjoyable to exercise; it is enjoyable to be motivated… You are developing a regular routine or habit of exercise … feeling better and better each day.

You will take some time each day to get some exercise. You will begin to exercise for 30 minute each day … You will find that you enjoy exercising … more and more each day. Each time that you finish exercising, you will enjoy it more and more. Each morning when you get up you will do some light yoga exercises. Each day you will notice that you are becoming more and more limber. As you see yourself becoming more flexible, you will want to practice yoga more and more. You will take some time out of each day to do some exercise, even if it just taking a 15-minute walk. Every time you have the option, you will walk somewhere rather than drive. You will find yourself more active and wanting to do more. EEach day you are finding ways of increasing your exercising whether it be by walking more, taking the stairs rather than the elevator, or even the occasional squat thrusts or setups. Your metabolism is increasing and you are becoming healthier.

Now take note of how proud you feel … so confident … full of energy … visualize yourself at your goal weight … you are confident and attractive …

Right now take a deep breath ... hold it for a moment of time ... exhale ... and relax. All of the suggestions I have given you are now strongly implanted and are part of you and will remain with you for as long as they are useful to you. In fact, with every breath you take... with every beat of your heart...the suggestions are becoming stronger and stronger...and are becoming your new habits for health...They become stronger and stronger ...and are your new habits for health.

1. Posthypnotic Suggestions:

From now on, it is going to be very easy for you to become hypnotized whenever you want to. We are going to establish a cue that will allow you to be hypnotized instantly. From now on, the words, hypnosis now, will be a signal for you to enter hypnosis. But it will only work when you say these words, either to yourself or out loud, and when you want to become hypnotized. When you want to enter hypnosis and say the words, hypnosis now, you will immediately become deeply engrossed in the hypnotic experience. But it won’t happen if someone else says these words. If you hear these words in normal conversation, they will have no effect at all.

And it won’t work if you do not wish to experience hypnosis. But if you say, hypnosis now, and if you are ready to be hypnotized, you will be able to enter hypnosis immediately. This way you will be able to use hypnosis whenever you like. You could use it before or during eating and after to return to your special place, that place of comfort and peace and to reinforce all the suggestions.

1. Alerting:

I am going to count backward from five, and with each count you are going to become more and more alert and energized. At the count of one, you can open your eyes. At zero, you will be fully alert and wide awake, feeling better than you did when we began. Five...four...feeling more and more alert...three...feel the energy flowing into you...two...more awake... one...open your eyes...zero...wide awake.

Control Audio file

Hello! I am Myrna Saadeh, clinical hypnotherapist.

When listening to this recording, you should only be listening to it in the morning and at night, when you are safe, and no one and nothing will disturb you.

You will be receiving positive suggestions that will create positive lifestyle changes and help you lose weight.

Never play this recording while you’re driving. Thank you and enjoy!

1. Induction:

Please make yourself comfortable...Close your eyes and let yourself relax... Take a few, slow, deep breaths and notice that as you exhale, you can feel yourself becoming more and more relaxed. You can continue to relax, as I speak to you. And each time you exhale, you can feel yourself becoming more and more relaxed...more and more relaxed. Soon you will experience hypnosis. I want to assure you that no matter how deeply hypnotized you become, you will remain in complete control. You will stay in control, even when very deeply involved in the experience of hypnosis. I will make suggestions, and you will experience those suggestions. You will find it very easy to experience what I'm telling you than you ever thought possible. So the choice is always yours, and it’s safe to enter hypnosis now, as you allow yourself to relax more and more. .

As I speak, you can feel yourself becoming more relaxed. But no matter how relaxed you become, you will hear my voice, and you will be able to respond to my suggestions. If you become uncomfortable, or need to pause or stop for whatever reason, you can readjust your body and make yourself comfortable again, and that won’t get in the way of your experience.

I am going to give you some suggestions that will make this a permanent change in your living. These suggestions are going to take complete and thorough effect upon the deepest part of your subconscious mind. They will remain there forever, and become a permanent part of every cell of your brain and body. You are going to be surprised and amazed just how effective these suggestions are going to be and how much they will become a part of your everyday life, giving you a brand new pattern, brand new thoughts, a brand new method of action, to make you successful in releasing all those extra kilograms.

1. Suggestions:

You will be a new person, in a new form, with new eating habits. You are going to enjoy life. You will eat only when you have physiological needs for food and no other time, for the rest of your life.

From this point forward, starting right now ... you will no longer have the urge to snack between meals. All desire to eat fattening, heavy, rich foods is draining away. The inclination to eat such foods is leaving you now ... and is becoming like a distant memory. It is simply a past experience ... it has no effect on you now. You no longer have the desire or appetite to get a late night snack; because you are already full... you are completely satisfied by a normal well balanced meal. You will no longer overeat at mealtime, because moderate meals will more than satisfy your hunger. And the flavor and tastes of food will be so sharp that your appetite will be satisfied, as it has never been before. The enhanced taste of food will fill you up quickly and satisfy your hunger. The delicate flavor of each bite of food will be more enjoyable than before. You enjoy the taste and fragrance of your food more than ever before. You will feel so much better, so much healthier, so much happier and so much more energetic without an uncomfortably over filled stomach.

Each time you are tempted to eat or drink anything that you know is wrong for you, you will say “no” and stick by it, because the rewards of enjoying your meals and taking care of yourself are more important to you than eating the wrong foods.

Now imagine your MOST IMPORTANT BENEFITS SO VIVIDLY that you feel as though you already enjoy success. Imagine your attitude of gratitude … and be glad. [Slowly] Imagine your benefits … SO VIVIDLY that you FEEL as though you already enjoy SUCCESS! If you choose these benefits for yourself, then indicate that choice right now by moving one of your fingers.

You have used your power of choice to choose your benefits. The terms are so simple. You simply use that same power of choice to choose what goes into your mouth … when, where, and how much. You decide what goes into your mouth … when, where, and how much. And you have an increasing satisfaction from the right amounts of those foods which help you reach your ideal, healthiest body weight. And you make wise choices about your health and eating habits. Also, whenever you choose water or a non-caloric beverage to satisfy in between meal snack urge, you are TOTALLY satisfied – physically, mentally, and emotionally, because YOU CHOOSE …

You LOVE your power of choice. And like a muscle that’s used becomes stronger with use, your power of choice becomes stronger with use. The benefits are so satisfying and you love your power of choice.

Also, whenever you eat, you eat SLOWLY enough to … ENJOY the FLAVOR of each bite. And when you have had enough food to give your body nourishment, you are satisfied - physically, mentally, and emotionally … and every day it becomes easier for you to be TOTALLY self-motivated to do those things that help you reach your ideal body weight, because you love the benefits.

You eat … SLOWLY enough to … LISTEN to your body and FEEL your satisfaction. You allow your physical and emotional appetites to harmonize. And when you have had enough to eat, you simply take your thumb or finger and do a PUSH AWAY. Simply push your plate even so slightly away from you – pushing away the excess food and the excess kilograms – kilogram after kilogram. You release the excess food and you release the excess kilograms - kilogram after kilogram – until you reach your ideal body weight.

Eating well balanced meals will increase your energy. And with your new lighter figure, you will find that your energies have increased and you will feel stronger and healthier as you approach and achieve your ideal weight and figure. Never again will you eat because you are nervous, tense, bored, frustrated, depressed or angry.

The key to achieving your fitness goals is to get excited about it. And from this moment on you are totally excited about the new you that you are creating. You now focus on the end results of what you would like to look like and how you feel as the goal you. Visualize yourself as the goal you already and realize what it took to get you here. Excitement, exercise and motivation. You are very eager to attain this goal and each day you wait in anticipation for the moment to come that you can begin exercising knowing that you are becoming the goal you each day. It is now enjoyable to exercise; it is enjoyable to be motivated… You are developing a regular routine or habit of exercise … feeling better and better each day.

You will take some time each day to get some exercise. You will begin to exercise for 30 minute each day … You will find that you enjoy exercising … more and more each day. Each time that you finish exercising, you will enjoy it more and more. Each morning when you get up you will do some light yoga exercises. Each day you will notice that you are becoming more and more limber. As you see yourself becoming more flexible, you will want to practice yoga more and more. You will take some time out of each day to do some exercise, even if it just taking a 15-minute walk. Every time you have the option, you will walk somewhere rather than drive. You will find yourself more active and wanting to do more. EEach day you are finding ways of increasing your exercising whether it be by walking more, taking the stairs rather than the elevator, or even the occasional squat thrusts or setups. Your metabolism is increasing and you are becoming healthier.

Now take note of how proud you feel … so confident … full of energy … visualize yourself at your goal weight … you are confident and attractive …

1. Post Suggestions

Right now take a deep breath ... hold it for a moment of time ... exhale ... and relax. All of the suggestions I have given you are now strongly implanted and are part of you and will remain with you for as long as they are useful to you. In fact, with every breath you take... with every beat of your heart...the suggestions are becoming stronger and stronger...and are becoming your new habits for health...They become stronger and stronger ...and are your new habits for health.

1. Alerting

In few moments, you can open your eyes, feeling wide awake and refreshed. open your eyes..
