# Supplementary material for: The use of audio self-hypnosis to promote weight loss using the transtheoretical model of change: a randomized clinical pilot trial
Source: PeerJ. 2022 Dec 14;10:e14422. doi: 10.7717/peerj.14422 (PMC9758970; doi:10.7717/peerj.14422)
Supplement: Supplemental Information 2 [file peerj-10-14422-s002.pdf]

CONSORT 2010 checklist of information to include when reporting a randomised trial*

Section/Topic	Item No	Checklist item	Reported on page No
**Title and abstract**			
	1a	Identification as a randomised trial in the title	Lines 1-3
	1b	Structured summary of trial design, methods, results, and conclusions (for specific guidance see CONSORT for abstracts)	Lines 21-33
**Introduction**			
Background and objectives	2a	Scientific background and explanation of rationale	Lines 43-45; 58-59; 71-75
	2b	Specific objectives or hypotheses	Lines 79-82
**Methods**			
Trial design	3a	Description of trial design (such as parallel, factorial) including allocation ratio	Line 85 ; 136
	3b	Important changes to methods after trial commencement (such as eligibility criteria), with reasons	Not applicable
Participants	4a	Eligibility criteria for participants	Lines 101-108
	4b	Settings and locations where the data were collected	Lines 94-99
Interventions	5	The interventions for each group with sufficient details to allow replication, including how and when they were actually administered	Lines 86-90
Outcomes	6a	Completely defined pre-specified primary and secondary outcome measures, including how and when they were assessed	Lines 110-120; 161-168
	6b	Any changes to trial outcomes after the trial commenced, with reasons	Not applicable
Sample size	7a	How sample size was determined	Lines 150-159
	7b	When applicable, explanation of any interim analyses and stopping guidelines	
Randomisation:			
Sequence generation	8a	Method used to generate the random allocation sequence	Lines 137-139
	8b	Type of randomisation; details of any restriction (such as blocking and block size)	Lines 137
Allocation concealment mechanism	9	Mechanism used to implement the random allocation sequence (such as sequentially numbered containers), describing any steps taken to conceal the sequence until interventions were assigned	Lines 138-140
Implementation	10	Who generated the random allocation sequence, who enrolled participants, and who assigned participants to interventions	Lines 138-140
Blinding	11a	If done, who was blinded after assignment to interventions (for example, participants, care providers, those	Lines 141

		assessing outcomes) and how	
	11b	If relevant, description of the similarity of interventions	
Statistical methods	12a	Statistical methods used to compare groups for primary and secondary outcomes	Lines 163-168
	12b	Methods for additional analyses, such as subgroup analyses and adjusted analyses	Not applicable
**Results**			
Participant flow (a diagram is strongly recommended)	13a	For each group, the numbers of participants who were randomly assigned, received intended treatment, and were analysed for the primary outcome	Lines 171-177
	13b	For each group, losses and exclusions after randomisation, together with reasons	Lines 171-177
Recruitment	14a	Dates defining the periods of recruitment and follow-up	Lines 171-173
	14b	Why the trial ended or was stopped	not applicable
Baseline data	15	A table showing baseline demographic and clinical characteristics for each group	Lines 174
Numbers analysed	16	For each group, number of participants (denominator) included in each analysis and whether the analysis was by original assigned groups	Figure 1
Outcomes and estimation	17a	For each primary and secondary outcome, results for each group, and the estimated effect size and its precision (such as 95% confidence interval)	Lines 178-182
	17b	For binary outcomes, presentation of both absolute and relative effect sizes is recommended	
Ancillary analyses	18	Results of any other analyses performed, including subgroup analyses and adjusted analyses, distinguishing pre-specified from exploratory	Lines 179-180
Harms	19	All important harms or unintended effects in each group (for specific guidance see CONSORT for harms)	
**Discussion**			
Limitations	20	Trial limitations, addressing sources of potential bias, imprecision, and, if relevant, multiplicity of analyses	Lines 215-221
Generalisability	21	Generalisability (external validity, applicability) of the trial findings	Lines 215-221
Interpretation	22	Interpretation consistent with results, balancing benefits and harms, and considering other relevant evidence	Lines 203-210
**Other information**			
Registration	23	Registration number and name of trial registry	Line 33 Line 35
Protocol	24	Where the full trial protocol can be accessed, if available	
Funding	25	Sources of funding and other support (such as supply of drugs), role of funders	Line 35

*We strongly recommend reading this statement in conjunction with the CONSORT 2010 Explanation and Elaboration for important clarifications on all the items. If relevant, we also recommend reading CONSORT extensions for cluster randomised trials, non-inferiority and equivalence trials, non-pharmacological treatments, herbal interventions, and pragmatic trials. Additional extensions are forthcoming: for those and for up to date references relevant to this checklist, see www.consort-statement.org.
