# Supplementary material for: The use of audio self-hypnosis to promote weight loss using the transtheoretical model of change: a randomized clinical pilot trial
Source: PeerJ. 2022 Dec 14;10:e14422. doi: 10.7717/peerj.14422 (PMC9758970; doi:10.7717/peerj.14422)
Supplement: Supplemental Information 4 [file peerj-10-14422-s004.pdf]

Research File available

S-Weight and P-Weight questionnaires (Spanish and English version)

April 2016
DOI: 10.13140/RG.2.1.3578.6648

Ana Andrés

Overview Stats Comments Citations References •••

Description

This file contains the Stages and Processes of Change questionnaires for Weight Management (S-Weigt and P-Weight) in its Spanish and English versions. These questionnaires have been already validated in Spanish and UK samples. Feel free to use them with research purposes and be careful when using them in your clinical sample (no published data is available yet on specific populations). Please, contact me if you have any questions, comments, or suggestions. If you are willing to apply them, please let me know, I will be more than happy to help you!
