# Supplementary material for: The use of audio self-hypnosis to promote weight loss using the transtheoretical model of change: a randomized clinical pilot trial
Source: PeerJ. 2022 Dec 14;10:e14422. doi: 10.7717/peerj.14422 (PMC9758970; doi:10.7717/peerj.14422)
Supplement: Supplemental Information 6 [file peerj-10-14422-s006.pdf]

ClinicalTrials.gov PRS
Protocol Registration and Results System

ClinicalTrials.gov Protocol Registration and Results System (PRS) Receipt
Release Date: July 20, 2021

ClinicalTrials.gov ID: NCT04247568

Study Identification

Unique Protocol ID: SBS-2019-0220

Brief Title: Hypnosis and States of Change to Promote Weight Loss

Official Title: The Use of Audiotaped Hypnosis in Promoting Weight Loss Using the Transtheoretical Model of Change

Secondary IDs:

Study Status

Record Verification: July 2021

Overall Status: Active, not recruiting

Study Start: December 4, 2019 [Actual]

Primary Completion: December 30, 2021 [Anticipated]

Study Completion: December 30, 2022 [Anticipated]

Sponsor/Collaborators

Sponsor: American University of Beirut Medical Center

Responsible Party: Principal Investigator
Investigator: Jumana Antoun [jumana antoun]
Official Title: Associate Professor of Clinical Family Medicine
Affiliation: American University of Beirut Medical Center

Collaborators:

Oversight

U.S. FDA-regulated Drug: No

U.S. FDA-regulated Device: No

U.S. FDA IND/IDE: No

Human Subjects Review: Board Status: Approved
Approval Number: SBS-2019-0220
Board Name: Institutional Review Board
Board Affiliation: American University of Beirut
Phone: 961135000
Email: irb@aub.edu.lb
Address:

American University of Beirut

Data Monitoring: No

FDA Regulated Intervention: No

Study Description

Brief Summary: Complementary and alternative therapies for weight loss treatment may be effective. There are few studies showing promise of the use of hypnosis in weight-reduction programs; however, there are lots of bias and more rigorous rials are needed to establish the relationship between hypnosis and weight management. Furthermore, the effect of hypnosis may not be directly related to weight loss but rather on behavioral change. On the basis of the trans theoretical model of change, we hypothesized that audio taped hypnosis would facilitate greater movement through the stages of change toward weight loss as compared to general advice.

OBJECTIVE: The primary aim of this study is to assess the ability of audio taped hypnosis to promote weight loss through its effect on the stages and the processes of change as defined by the Trans theoretical Model of change.

DESIGN: Randomized controlled trial. SETTING: American University of Beirut Medical Center. PARTICIPANTS: Adults with overweight and obesity will be recruited if they had previous attempt to lose weight, are planning to lose weight within the next 6 months or are not satisfied with the results of their current weight loss plan.

INTERVENTIONS: This research will be triple blinded randomized placebo controlled trial. The intervention group will be listening to a hypnotic audio-file on a USB and the control group will be listening to a placebo audio-file on a USB . The hypnotic audio-file will consist of a 20 minutes recording prepared by an experienced hypnotist and the control audio-file will consist of a 20 minutes recording with direct messages targeting lifestyle modification. Follow up visits will take place at 21 days, 3 months, 6 months and 12 months following the intervention to assess for any change in participant's readiness to lose weight.

MAIN OUTCOME MEASURES: The primary outcome will be the difference between the groups in acquiring at least one stage change (upward) as defined by the S-weight from baseline to 3 weeks, 6 months and 12 months post intervention. The secondary outcomes include difference in the mean score of any item of the processes of change between hypnosis audio-file and control audio-file, weight in kg at 3 weeks, 6 and 12 months as compared to baseline weight, decrease in waist circumference in cm as compared to baseline at 6 and 12 months between the hypnosis and control groups, exploring factors that may affect any of the primary and secondary outcomes such as gender, age, educational status, baseline BMI, PHQ2.

Detailed Description:

Conditions

Conditions: Obesity
Weight Loss

Keywords: Weight loss
Hypnosis
Adults
Behavior change

Study Design

Study Type: Interventional

Primary Purpose: Treatment

Study Phase: N/A

Interventional Study Model: Parallel Assignment

Number of Arms: 2

Masking: Double (Participant, Outcomes Assessor)

Allocation: Randomized

Enrollment: 60 [Actual]

Arms and Interventions

Arms	Assigned Interventions
Experimental: Hypnosis	Listening to an audiotape<br>The participants listen to a audiofile that consists of hypnosis
Placebo Comparator: Control	Listening to an audiotape<br>The participants listen to a audiofile that consists of hypnosis

Outcome Measures

Primary Outcome Measure:

1. Acquiring at least one stage change (upward) as defined by the S-weight
   the difference between the groups in acquiring at least one stage change (upward) as defined by the S-weight from baseline to 3 weeks, 6 months and 12 months post intervention.

   [Time Frame: 21 days, 3 months, 6 months and 12 months]

Secondary Outcome Measure:

2. Weight change
   difference in the weight between hypnosis audio-file and control audio-file, at 3 weeks, 6 and 12 months as compared to baseline weight

   [Time Frame: 21 days, 3 months, 6 months and 12 months]
3. difference in the mean score of any item of the processes of change
   difference in the mean score of any item of the processes of change between hypnosis audio-file and control audio-file, weight in kg at 3 weeks, 6 and 12 months as compared to baseline

   [Time Frame: 21 days, 3 months, 6 months and 12 months]
4. Change in waist circumference in cm as compared to baseline
   decrease in waist circumference in cm as compared to baseline

   [Time Frame: 6 and 12 months]

Eligibility

Minimum Age: 18 Years

Maximum Age: 64 Years

Sex: All

Gender Based: No

Accepts Healthy Volunteers: Yes

Criteria: Inclusion Criteria:

- BMI 25 kg/m2 and above, aged 18 to 64 years, able to give written informed consent, fluent in English, all men and women not planning on getting pregnant within the next 12 months, with previous attempts to lose weight, planning to lose weight within the next 6 months or not satisfied with the results of their current weight loss plan.

Exclusion Criteria:

- individuals diagnosed with a psychotic disorder, currently on an antipsychotic medication, pregnant women, women planning on getting pregnant during the study period, not planning on losing weight within the next 6 months, not meeting the inclusion criteria or those who are satisfied with their weight loss progress, and illiterate.

Contacts/Locations

Central Contact Person: Jumana Antoun, MD
Telephone: +9613486509
Email: ja46@aub.edu.lb

Central Contact Backup:

Study Officials:

Locations: **Lebanon**
Jumana Antoun
Hamra, Beyrouth, Lebanon, 110236
Contact: Jumana Antoun 03486509 ja46@aub.edu.lb

IPDSharing

Plan to Share IPD: No

References

Citations:

Links:

Available IPD/Information:
